# Strong coupling of double resonance designs and epsilon-near-zero modes for mode-matching enhancement of second-harmonic generation

**DOI:** 10.1515/nanoph-2025-0382

**Published:** 2025-10-23

**Authors:** Ai-Yin Liu, Chun-Hsiang Tseng, Kuang-I Lin, Hui-Hsin Hsiao

**Affiliations:** Graduate Institute of Photonics and Optoelectronics, 33561 National Taiwan University, Taipei, 10617, Taiwan; Department of Engineering Science and Ocean Engineering, National Taiwan University, Taipei, 10617, Taiwan; Core Facility Center, National Cheng Kung University, Tainan, 70101, Taiwan; Department of Engineering Science and Ocean Engineering and Graduate Institute of Photonics and Optoelectronics, National Taiwan University, Taipei, 10617, Taiwan

**Keywords:** split-ring resonators, epsilon-near-zero materials, strong coupling, nonlinear optics, second-harmonic generation

## Abstract

We investigate the coupling effects between a double-resonance split-ring resonators (SRRs) design and the epsilon-near-zero (ENZ) guided mode of ultrathin indium tin oxide (ITO) films on second-harmonic generation (SHG). The optimized SRRs with an aspect ratio of 0.3 support a magnetic dipole (MD) resonance within the ENZ regime of ITO and a higher-order resonance at the SH frequency to achieve mode matching under cross-polarized excitation. The SRR-ITO coupled system (as opposed to the nanorod-ITO coupled system) was found to perform constructive (destructive) polarization interference between the nonlinear polarization currents at the upper hybridized modes (ω^+^) and the linear electric field at SH frequency (2ω^+^), resulting in a 1218-fold SHG enhancement outperformed than that of the nanorod-ITO coupled system, as predicted by overlap integral analysis. The measured SHG conversion efficiency for the SRR-ITO coupled system exceeds 10^−7^ at an excitation wavelength of 1,320 nm, corresponding to a one-order (two-order) of magnitude enhancement compared to the nanorod-ITO coupled system (Au/ITO film). These findings highlight the potential of the proposed hybrid metasurfaces for efficient cross-polarized nonlinear signal generation, paving the way for advanced applications such as light sources, modulators in integrated photonic circuits, and biological sensing.

## 1 Introduction

Second-harmonic generation (SHG) has attracted considerable interest due to its wide-ranging photonic applications in biological sensing [1], [2], vacuum ultraviolet light source [3], [4], and high-resolution microscopy [5], [6]. A variety of plasmonic nanostructures that support magnetic resonances [7], [8], [9], [10], Fano resonances [11], [12], and surface lattice resonances [13], [14], [15], [16] have been demonstrated to support strongly localized electric field at the fundamental frequency for amplifying SHG. However, as noble metals such as gold and silver exhibit centrosymmetric crystal lattices, SHG from plasmonic systems is prohibited in the bulk volume and predominantly originate from the metallic surfaces where centro-symmetry is broken. To improve the conversion efficiency, some studies has been indicated that the high degree of the asymmetric spatial variation for the inducing electromagnetic fields play a role in affecting the intensity of the generated second-harmonic (SH) signals [17], [18], [19]. In addition, doubly or multi-resonance designs cover the wavelengths involved in the nonlinear process [13], [20], [21], [22] have been demonstrated as an effective strategy to maximize the nonlinear effects.

The most straightforward approach for double-resonance designs is to integrate multiple resonators into one single metasurface, allowing the spectral positions of resonances to be freely adjusted through the geometrical tuning of each resonator and their mutual coupling [23], [24]. For example, aluminum antennas with three different arm lengths have been designed to exhibit resonances at both the fundamental and SH frequencies, and a maximal SHG enhancement is found to achieve when the quadrupole-dipole coupling occurs to allow efficient dipole SH emission [24]. In addition, the combination of V-shaped and nanorod antennas has been shown to support double resonances while ensuring spatial mode overlap [25]. Another class of structures employs a sandwich configuration, consisting of plasmonic nanocubes atop a dielectric spacing layer and a back metallic reflector, which achieves mode-matched SHG through gap-plasmon modes and the localized surface plasmon resonance of the nanocube [26].

**Figure 1:**
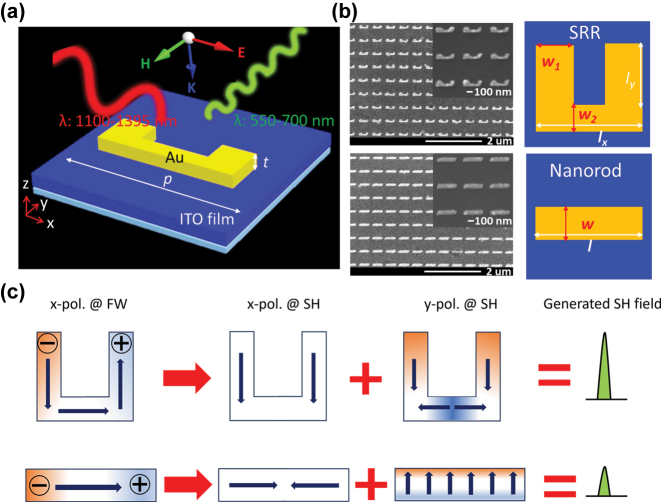
Double-resonance hybrid metasurfaces. (a) Schematic illustration of nonlinear SRR-ITO coupled system. (b) Left: SEM images of the SRR-ITO coupled system (top) and the nanorod-ITO coupled system (bottom). Right: Geometrical parameters of the SRRs (top) and nanorods (bottom). (c) Illustration of SHG enhancement using double-resonance SRR designs. The color maps represent the linear electric field distributions, while the indigo arrows indicate the directions of the surface currents. The middle row (*x*-pol. @ SH) corresponds to the nonlinear currents generated by the fundamental mode (*x*-pol. @ FW). The resulting nonlinear signal interacts with a higher-order mode (*y*-pol. @ SH), leading to the final SHG response.

Later, the development of the overlap integral further indicates that maximizing nonlinear interactions in nanoscale systems requires not only a spatial overlap of the excited local field at both the fundamental and SH frequencies but also constructive polarization interference between them. To achieve this, a metal–insulater–metal (MIM) sandwich structure incorporating an epsilon-near-zero (ENZ) material as a spacer has been studied, enabling spatial and constructive overlap between fundamental wave (FW) and SH polarizations through different orders of gap-plasmon modes [22]. The effectiveness of the overlap integral in predicting SHG behavior for nanostructures becomes even more evident when optimizing the SHG efficiency of split-ring resonators (SRRs) [27]. The SRR designs are well-known structures to be able to generate strong magnetic-dipole (MD) resonances when the polarization of the incident light is parallel to the base arm of the SRR structure (i.e., *x*-direction in Figure 1a) [28]. The generated magnetic field associated with the MD resonance causes the electrons in the base arm are not only driven by the incident electric field but also experienced magnetic Lorentz force simultaneously. According to the hydrodynamic model, this Lorentz force supplies the contribution of SHG signals polarized orthogonally to the excitation (i.e., *y*-direction in Figure 1a) [27], [28], [29], [30]. By carefully tailoring the geometry of SRRs, a higher-order resonance is possible to be designed at the SH frequency under the excitation of an orthogonal polarized light. Previous study has demonstrated that a maximal SH does not occur for SRRs with the severe asymmetry but for intermediate morphology through the overlap integral theoretical prediction and was validated by the experimental results [27].

To further boost up the SHG performance of nonlinear plasmonic metasurfaces, previous studies have incorporated materials with high nonlinear susceptibility such as indium tin oxide (ITO) [31], [32], [33], [34], lithium niobate [35], [36], HfO_2_-based ferroelectric [22], [37], and TiO_2_ [38] into nanostructures. Among these, ITO films exhibit a second-order susceptibility $\chi^{\left(2\right)}$ up to eight orders of magnitude higher than that of gold [34]. In addition, ITO supports a bulk-plasma mode with substantial electric field confinement inside the cavity to intensify the nonlinear response near its ENZ wavelength [39], [40], [41], [42]. Thus, strong coupling between plasmonic or dielectric nanostructures and the ENZ mode of ultrathin ITO films has been widely investigated for enhancing Kerr effects [31], [32], [43], [44], [45], as well as for nonlinear signal up-conversion (SHG [33], [34] or THG [46]) and down conversion [47]. While most prior demonstrations focus on overlapping a single resonant mode with the ENZ wavelength of ITO, we design a double-resonance Au SRR array that supports a MD mode within the ENZ region of the ITO layer and a higher-order mode at the SH frequency under cross-polarization excitation. The MD-ENZ interaction in the SRR-ITO coupled system gives rise to two new hybridized modes (ω^+^ and ω^−^) through strong coupling. More importantly, we explicitly examined the interference between the nonlinear polarization currents and the linear electric field at SH frequency in the coupled systems and found that constructive (destructive) polarization interference is achieved for the SRR-ITO (nanorod-ITO) coupled system under normal incidence, in contrast to previous MIM sandwich structures that requires oblique incidence to simultaneously excite the first- and second-order gap-plasmon modes for spatial and constructive mode matching [22] (see detailed comparison in Table 1 in supplementary material). The overlap integral analysis shows a 1218-fold SHG enhancement in the SRR-ITO coupled system compared to that of the nanorod-ITO coupled system. The measured SHG conversion efficiency of the SRR-ITO coupled system exceeds 2.3 × 10^−7^ at the wavelength of 1,320 nm, corresponding to one order of magnitude (two orders of magnitude) larger than the measured SH signals for nanorod-ITO coupled system (Au/ITO film). These findings underscore the potential of hybrid SRR-ITO metasurfaces for highly efficient SHG, while their cross-polarization characteristics between the FW and SH signals further paves the way for advanced nonlinear optical devices.

## 2 Results and discussions

Figure 1a and b depicts the unit cell of the hybrid metasurface comprising Au SRR arrays on a 40-nm-thick ITO film, with a glass substrate beneath the structure. The SRRs are arranged in a square lattice with a period (*P*) of 450 nm. Each SRR consists of two arms with the width (*w*_*1*_) of 80 nm and the length *l*_*y*_, alongside a bottom arm with a width (*w*_*2*_) of 40 nm and a length *l*_*x*_. The thickness (*t*) of these Au nanostructures is set to be 50 nm. In addition, to ensure an effective coupling between the plasmonic resonance of Au nanostructures and the bulk-plasmon mode of ITO film, the resonant modes for Au nanostructures in the absence of ITO films should fall within the ENZ region. The ENZ region of ITO film, defined as the range where the absolute value of the real part of permittivity is below 1 (i.e., $-1\leq \text{Re}\left\{\varepsilon \right\}\leq 1$ ), falls between the wavelength range of 1,190 and 1,512 nm [46].

Figure 1c illustrates the design concept of double-resonance SRRs under cross-polarized excitation. For the incidence of *x*-polarized light, the induced circular surface currents of SRRs leads to a MD resonance at the fundamental frequency $\left(\omega \right)$ (x-pol. @FW in Figure 1c). According to the hydrodynamic model [48], the magnetic field of the MD resonance will lead a Lorentz force acting on free electrons, thus generating SHG signals polarized along the *y*-direction (x-pol. @SH in Figure 1c). Meanwhile, a higher-order resonance is designed to be excited at SH frequency $\left(2\omega \right)$ under the illumination of *y*-polarized light (y-pol. @SH in Figure 1c). Thus, the constructive interference between the induced SHG and the higher-order resonance at the SH frequency is beneficial for the enhancement of SHG.

To maximize the SHG of double-resonance SRRs in the uncoupled system, we first employed the overlap integral to determine the optimal ARs of the SRR structures. Here, the AR is defined as the ratio of the vertical length to the total length of the SRR nanostructure, given by AR = $\frac{2l_{y}}{l_{x}+2l_{y}}$ . The AR was varied from 0.19 to 0.33 by adjusting *l*_*x*_ from 380 to 280 nm. An AR of 0 corresponds to Au nanorod arrays (Figure 1b). All samples were designed with the same surface area to ensure a fair comparison. Numerical simulations were conducted using the COMSOL Multiphysics software, based on the finite element method, to calculate both the linear and nonlinear model in the frequency domain. The permittivity of the 40-nm-thick ITO film was acquired by the measurement from the spectroscopic ellipsometry [46]. The refractive index of the glass substrate was set to 1.5, and the dielectric function of gold was adopted from Johnson and Christy [49].

**Figure 2:**
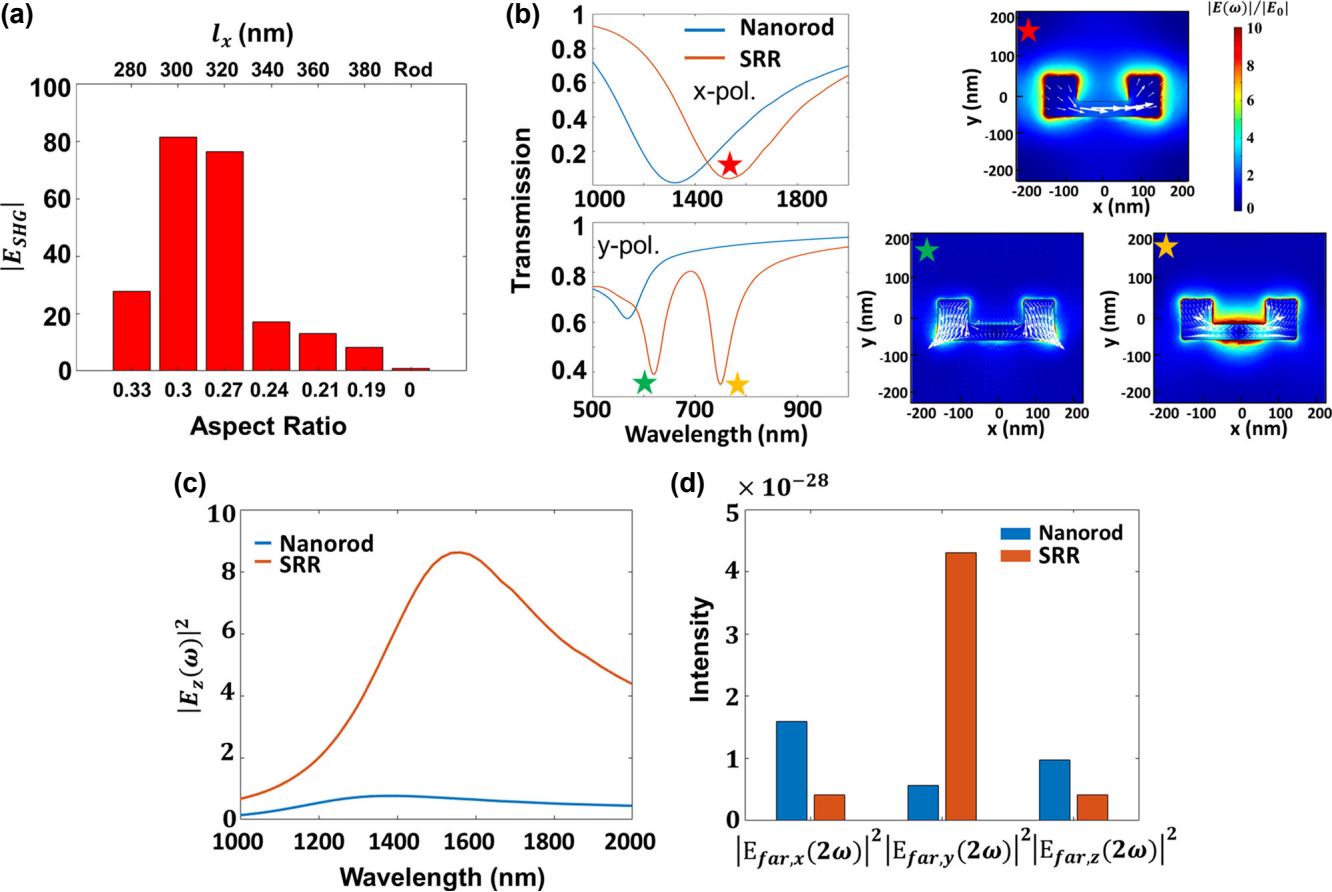
Optimization of the ARs for SRRs in uncoupled systems. (a) Evaluated $\left|E_{\text{SHG}}\right|$ as a function of the AR for SRRs, where AR is defined as $\frac{2l_{y}}{l_{x}+2l_{y}}$ . (b) Simulated transmission spectra of the SRR (*l*_*y*_ = 65 nm and *l*_*x*_ = 300 nm) and nanorod (*l* = 350 nm and *w =* 65 nm) arrays on a glass substrate under normal incidence with *x*-polarized light (top panel) and *y*-polarized light (bottom panel). The red star denotes the spectral position of the MD resonance, while the yellow and green starts indicate the spectral positions of the higher-order resonances. Insets display the corresponding electric field distributions for each resonance mode, with the white arrows indicating the surface current distributions. (c) Integration of ${\left|E_{z}\left(\omega \right)\right|}^{2}$ over the bottom plane of the SRRs (red curve) and nanorods (blue curve). (d) The *x*, *y*, and *z* components of nonlinear far-field intensity for arrays of nanorods (blue bars) and SRRs (orange bars), respectively.

The overlap integral between the nonlinear polarization and the linear electric field at SH can be expressed as: [27]

(1) $$\left|E_{\text{SHG}}|\propto |\iint \text{P}\left(2\omega \right)\cdot E\left(2\omega \right)dA\right|$$

where |*E*_SHG_| represents the amplitude of electric field for SHG emission in the far field, $P\left(2\omega \right)$ denotes the nonlinear polarization at the SH frequency under *x*-polarized excitation, and $E\left(2\omega \right)$ corresponds to the linear electric field at the SH frequency under *y*-polarized excitation, owing to the cross-polarization property of the SRR structures. $P_{i}\left(2\omega \right)$ is obtained by applying the relation of $P_{i}\left(2\omega \right)=\underset{\text{ijk}}{\sum }\varepsilon_{0}\chi_{\text{ijk}}^{\left(2\right)}E_{j}\left(\omega \right)E_{k}\left(\omega \right)$ , where $\chi^{\left(2\right)}$ represents the second-order optical susceptibility of Au. The dominant $\chi^{\left(2\right)}$ elements for Au are $\chi_{\text{zzz}}^{\left(2\right)}=4.42\times 10^{-22}m^{2}/V$ , $\chi_{\text{zxx}}^{\left(2\right)}=\chi_{\text{zyy}}^{\left(2\right)}=4.94\times 10^{-21}\;m^{2}/V$ , and $\chi_{\text{xxz}}^{\left(2\right)}=\chi_{\text{yyz}}^{\left(2\right)}=\chi_{\text{yzy}}^{\left(2\right)}=\chi_{\text{xzx}}^{\left(2\right)}=1.17\times 10^{-21}\;m^{2}/V$ [34]. Thus, for Au nanostructures, the nonlinear polarization $P_{i}\left(2\omega \right)$ can be expressed as:

(2) $$P_{x}\left(2\omega \right)=2\varepsilon_{0}\chi_{\text{xzx}}^{\left(2\right)}E_{x}\left(\omega \right)E_{z}\left(\omega \right)$$

(3) $$P_{y}\left(2\omega \right)=2\varepsilon_{0}\chi_{\text{yyz}}^{\left(2\right)}E_{y}\left(\omega \right)E_{z}\left(\omega \right)$$

(4) $$\begin{array}{ll} P_{z}\left(2\omega \right) & =\varepsilon_{0}\left[\chi_{\text{zxx}}^{\left(2\right)}E_{x}\left(\omega \right)E_{x}\left(\omega \right)+\chi_{\text{zyy}}^{\left(2\right)}E_{y}\left(\omega \right)E_{y}\left(\omega \right)\right.\\ & \;+\left.\chi_{\text{zzz}}^{\left(2\right)}E_{z}\left(\omega \right)E_{z}\left(\omega \right)\right] \end{array}$$

where $E_{x}\left(\omega \right)$ , $E_{y}\left(\omega \right)$ , and $E_{z}\left(\omega \right)$ are the *x*, *y*, and *z* components of the electric field at the FW frequency, respectively. Figure 2a presents the calculated $\left|E_{\text{SHG}}\right|$ obtained via the overlap integral, for SRRs with varying ARs. The maximal $\left|E_{\text{SHG}}\right|$ occurs at an AR of 0.3, corresponding to 81.4-fold enhancement compared to that of the nanorods. Even at an AR of 0.19, the SRRs still exhibit a 8.4-fold enhancement in $\left|E_{\text{SHG}}\right|$ compared to that of the nanorods, owing to the absence of resonances at the SH frequency for the nanorods.

Figure 2b displays the simulated transmission spectra for the optimized SRR with *l*_*y*_ = 65 nm and *l*_*x*_ = 300 nm (AR = 0.3) under normal incidence of *x*- and *y*-polarized light, respectively. Under *x*-polarized light excitation, a broadband transmission dip appears at *λ* = 1,537 nm (red curve) with a full width at half maximum (FWHM) of 400–500 nm. This broadband resonance provides a large spectral overlap with the ENZ region of the ITO film. The near-field distribution marked by the red star reveals that it corresponds to the MD resonance induced by circular surface currents (white arrows) within the SRRs (Figure 2b). Meanwhile, the simulated transmission spectra of the optimized SRRs (red curve) under *y*-polarized excitation exhibit two distinct resonant dips at *λ* = 749 nm and 621 nm, respectively. Notably, the resonant mode at *λ* = 749 nm closely matches the SH frequency of the MD mode at *λ* = 1,537 nm, illustrating a double-resonance condition. Both the near-field distributions of the resonant modes at *λ* = 749 nm and *λ* = 621 nm, indicated by the yellow and green stars in Figure 2b, respectively, reveal the higher-order resonant feature. The variation of resonant modes for SRR structures with varying ARs under both *x*- and *y*-polarized light can be found in Figure S2 of supplementary material. In addition, Figure 2b shows the simulated transmission spectra of the nanorods with *l =* 350 nm and *w =* 65 nm for comparison. Upon *x*-polarized light illumination, one can observe a broad transmission dip at *λ* = 1,326 nm (blue curve), corresponding to the electric dipole (ED) resonance with the electric field primarily concentrated at both ends of the nanorods (Figure S2c in supplementary material). Different from SRRs, only one resonant mode at *λ* = 567 nm is observed for the nanorods under *y*-polarized excitation, which presents a large spectral deviation from the SH frequency of the ED mode at *λ* = 1,326 nm.

Next, we also performed a two-step nonlinear model based on perturbation theory [50]. First, the electric field at the FW frequency $\left(\text{E}\left(\omega \right)\right)$ is obtained using a linear electromagnetic model. This field is then used to compute the nonlinear polarization at SH frequency $P_{i}\left(2\omega \right)$ by applying the relation of $P_{i}\left(2\omega \right)=\underset{\text{ijk}}{\sum }\varepsilon_{0}\chi_{\text{ijk}}^{\left(2\right)}E_{j}\left(\omega \right)E_{k}\left(\omega \right)$ , where *χ* ^(2)^ represents the second-order optical susceptibility of Au. Using the obtained $P_{i}\left(2\omega \right)$ as a new source and solving the electrodynamic model at the SH frequency again, the electric field at the SH frequency, $\text{E}\left(2\omega \right)$ , is obtained. By applying the near-to-far-field transformation, the nonlinear electric field in the far field can be evaluated. Figure 2d compares the *x*, *y*, and *z* components of the far-field intensity at the SH frequency (i.e., ${\left|E_{\text{far,x}}\left(2\omega \right)\right|}^{2}$ , ${\left|E_{\text{far,y}}\left(2\omega \right)\right|}^{2},\text{and}\;{\left|E_{\text{far,z}}\left(2\omega \right)\right|}^{2}$ ) for the optimized SRRs and nanorods, respectively. It can be observed that the nanorods exhibit a dominant *x*-polarized far-field intensity at the SH frequency, whereas the SRRs produce *y*-polarized SH signals, in agreement with predictions from the hydrodynamic model. In addition, to preliminarily assess the influence of the SRRs and nanorods on the excitation of the bulk-plasmon mode via mode coupling, the normal component of the electric field intensity, ${\left|E_{z}\left(\omega \right)\right|}^{2}$ , at the bottom plane of Au nanostructures in the uncoupled system was integrated, as displayed in Figure 2c. The SRRs exhibit a 12-fold larger integrated field intensity compared to the nanorods.

**Figure 3:**
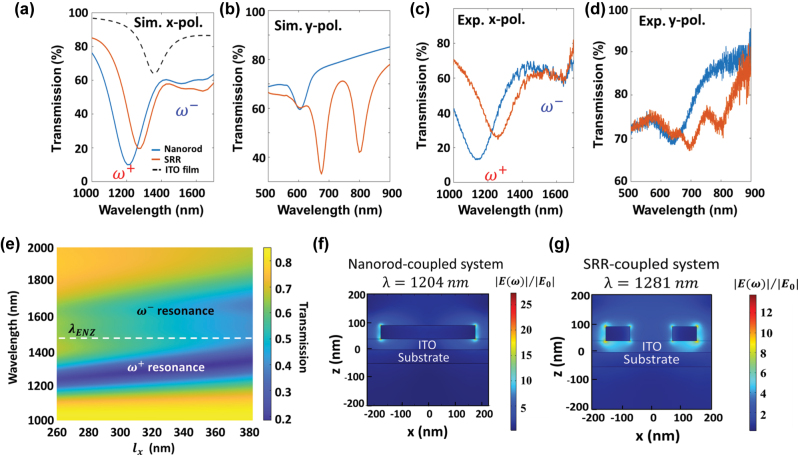
Optical properties of coupled systems. (a) Simulated transmission spectra for arrays of SRRs and nanorods on an ultrathin ITO film under the illumination of (a) *x*- and (b) *y*-polarized light, respectively. The dashed curve represents the transmission spectra of the bare ITO film at an oblique incidence angle of 50°. *ω* ^+^ and *ω* ^−^ indicate the spectral positions of upper and lower branches of the hybridized resonances, respectively. The corresponding measured transmission spectra under the incidence of (c) *x*- and (d) *y*-polarized light, respectively. (e) Simulated transmission map as a function of *l*_*x*_. The white dashed line indicates the ENZ wavelength (*λ*_ENZ_) of the ITO film. Electric field distributions at the respective *ω* ^+^ resonance for (f) the SRR-ITO system and (g) the nanorod-ITO system, respectively.

Figure 3a illustrates the calculated transmission spectra for the coupled systems with the insertion of a 40-nm-thick ITO film beneath both the nanorods (blue curve) and the optimized SRRs (red curve). The dashed curve represents the calculated transmission of the bare ITO film at an oblique incidence angle of 50°, featuring a prominent resonance dip at 1,360 nm, attributed to the bulk-plasma mode. The coupling between the MD mode of SRRs (the ED mode of nanorods) and the bulk-plasma resonance of the ITO film results in two new hybridized resonances (*ω* ^+^ and *ω* ^−^) with a Rabi splitting energy of 211 meV (285 meV). Figure 3e shows the transmission map of the SRR-ITO coupled system as a function of *l*_*x*_, revealing a pronounced spectral splitting into two hybridized resonances on either side of the ENZ wavelength (*λ*_ENZ_ = 1,360 nm). Figure 3f and g shows the electric field distributions of the SRR-ITO and nanorod-ITO coupled systems at the upper branch of the hybridized resonances ( $\left.\omega^{+}\right)$ , respectively. The near-field distributions reveal that the coupling between plasmonic resonances and the bulk-plasma mode leads to strong field confinement at both the surfaces of the metallic nanostructures and within the ITO cavity.

Since the critical coupling condition for a coupled system requires that the individual uncoupled mode has a comparable Q-factor, we first evaluated the total Q-factor of each resonant mode in the uncoupled system (*Q*_*tot*_) by using the relation 1/*Q*_tot_ = 1/*Q*_rad_ + 1/*Q*_abs_, where *Q*_rad_ and *Q*_abs_ represent the energy dissipation rates due to the radiation and absorption losses, respectively. The *Q*_tot_ for plasmonic nanostructures can be estimated by fitting the transmission spectra using the Fano line-shape equation (Figure S1e in supplementary material) [51],

(5) $$T_{\text{Fano}}={\left|a_{1}+ia_{2}+\frac{b}{\omega -\omega_{0}+i\gamma_{\text{tot}}}\right|}^{2}$$

where *a*_1_, *a*_2_, and *b* are the constants, *ω*_0_ is the central angular frequency of the resonant mode, and *γ*_*tot*_ denotes the total damping rate. Then, *Q*_*tot*_ for the MD resonance of the SRRs is determined by $Q_{\text{tot}}=\frac{\omega_{0}}{2\gamma_{\text{tot}}}$ and yields a value of 2.5. On the other hand, the *Q*_tot_ for the bulk-plasma resonance in the ITO film is primarily determined by the intrinsic material loss and was obtained from the eigenmode solver using COMSOL software, which extracts the complex eigenfrequency *ω*_eigen_​ and thus we can evaluate the Q-factor of the ENZ mode as

(6) $$Q_{\text{tot,ENZ}}=Q_{\text{abs,ENZ}}=\left|\frac{Re\left(\omega_{\text{eigen}}\right)}{2Im\left(\omega_{\text{eigen}}\right)}\right|$$

where the real part of *ω*_eigen_ determines the resonant frequency, and the imaginary part represents the damping rate and yields a value of 12. Therefore, the critical coupling in the SRR-coupled system is achieved when 1/*Q*_abs,ENZ_ = 1/*Q*_tot, SRRs_ is satisfied. The close value of *Q*_abs,ENZ_ and *Q*_tot,SRRs_ indicates that the SRR-ITO coupled system approaches the critical coupling condition. In addition, to verify strong coupling behavior between the MD resonance and the ENZ mode, we also calculate the coupling strength (*g)*, the damping rate of the SRRs (*γ*_SRRs_), and the damping rate of the ITO film (*γ*_ENZ_) based on the coupled mode theory [52] and found the SRR-ITO coupled system satisfies the strong coupling criteria with $g>\frac{\left|\gamma_{\text{SRRs}}-\gamma_{\text{ENZ}}\right|}{2}$ and $g>\frac{\sqrt{{\gamma_{\text{SRRs}}}^{2}+{\gamma_{\text{ENZ}}}^{2}}}{4}$ , where the extracted values are *g* = 141 meV, *γ*_SRRs_, = 267 meV, and *γ*_ENZ_ = 79 meV.

**Figure 4:**
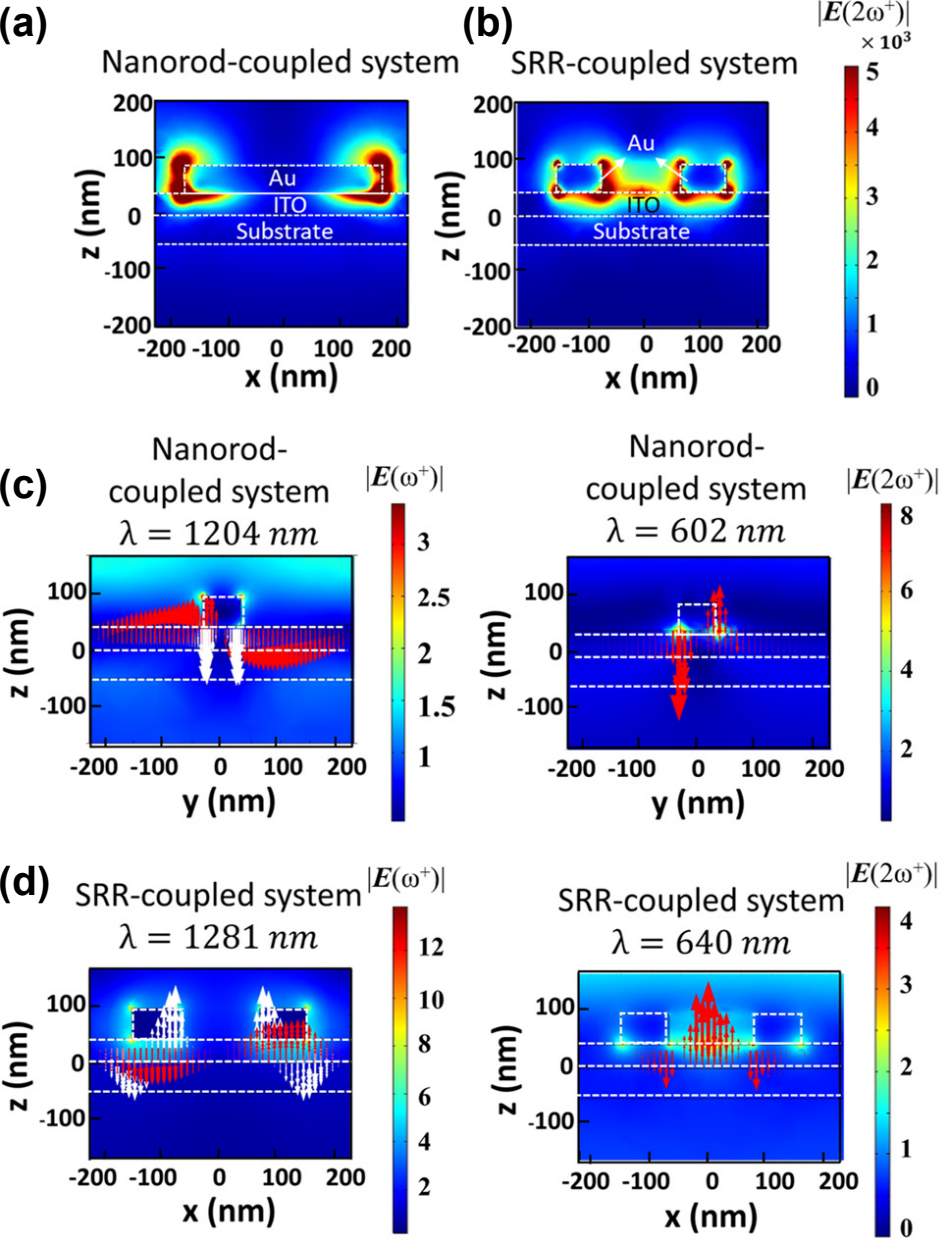
Nonlinear optical properties for the coupled systems. Nonlinear electric field at the SH frequency $\left|E\left(2\omega^{+}\right)\right|$ for (a) the nanorod-ITO and (b) the SRR-ITO coupled systems, respectively, calculated by the perturbation theory under an input power of 1 W. The linear electric-field and *E*_*z*_-field distributions for (c) the nanorod-ITO and (d) the SRR-ITO coupled systems, respectively. Red and white arrows in the left panel indicate the distributions of $E_{z}\left(\omega^{+}\right)$ and $E_{z}^{2}\left(\omega^{+}\right)$ under *x*-polarized excitation, respectively, and red arrows in the right panel indicate the distributions of *E*_*z*_(2*ω* ^+^) under *y*-polarized excitation. The color maps show the distributions of the electric fields at the *ω* ^+^ resonance $\left|\text{E}\left(\omega^{+}\right)\right|$ (left panel) and the corresponding SH frequency $\left|\text{E}\left(2\omega^{+}\right)\right|$ (right panel).

All samples were fabricated using standard electron beam lithography and thermal evaporation techniques. The scanning electron microscope (SEM) images of the fabricated structures are displayed in Figure 1b (see details in section S2 of supplementary material). Figure 3c presents the measured transmission spectra for the coupled system under normal incidence with *x*-polarized light, which agrees well with simulation results (Figure 3a) showing two new hybridized resonances (*ω* ^+^ and *ω* ^−^) with Rabi splitting energies of 328 meV for the nanorod-ITO coupled system and 224 meV for the optimized SRR-ITO coupled system. The slight broadening in the measured spectra may be attributed to surface roughness and dimensional deviations in the nanostructures. Interestingly, under normal incidence of *y*-polarized light, the simulated transmission spectra shows that the higher-order mode of the SRR-ITO coupled system at the shorter wavelength of 675 nm show good correspondence to the SH frequency of *ω* ^+^ hybridized mode (*λ* = 1,281 nm). Similarly, the higher-order mode of nanorod-ITO coupled system excited at *λ* = 613 nm also exhibit smaller spectral deviation with respect to the SH frequency of *ω* ^+^ hybridized mode (*λ* = 1,204 nm). The measured transmission spectra under *y*-polarized light also show good correspondence to simulation results (Figure 4b and d).

Next, the SHG performance for the coupled systems was analyzed via the aforementioned two-step nonlinear model, taking into account the bulk second-order susceptibility of $\chi_{\text{zzz}}^{\left(2\right)}$ = 1.8 × 10^−13^ m/V for ITO films [53]. Figure 4a and b illustrates the nonlinear electric field distributions at SH frequencies of the hybridized mode at *ω* ^+^ branch ( $\left.\left|E\left(2\omega^{+}\right)\right|\right)$ for the nanorod-ITO and the SRR-ITO coupled systems, respectively. Both coupled systems reveal that the nonlinear electric field is predominantly localized at the edges of the Au nanostructures and within the ITO cavities. The overlap integral was also used to analyze the magnitude of SHG enhancement for the coupled systems. According to Equation (1), the spatially overlapped enhanced fields at both the FW and SH frequencies must constructively add to maximize the nonlinear response. Since the $\chi_{\text{zzz}}^{\left(2\right)}$ of ITO films is approximately eight orders of magnitude larger than that of gold, our analysis focuses on the symmetry of fields between $E_{z}^{2}\left(\omega \right)$ under *x*-polarized excitation and *E*_*z*_(2ω) under *y*-polarized excitation. For the nanorod-ITO coupled system, the red arrows in Figure 4c indicate that the *z*-component of the electric field $\left(E_{z}\right.\left(\omega \right)$ ) exhibits odd symmetry at both *ω* ^+^ (left panel) and the corresponding SH frequency 2*ω* ^+^ (right panel), whereas the squared field $E_{z}^{2}\left(\omega \right)$ indicated by the white arrows displays even symmetry. The even symmetry of the square field at $\omega^{+}\left(E_{z}^{2}\left(\omega^{+}\right)\right)$ interacting with an antisymmetric $E_{z}\left(2\omega^{+}\right)$ -field results in a reduced $\left|E_{\text{SHG}}\right|$ . In contrast, as shown in Figure 4d, the $E_{z}\left(\omega^{+}\right)$ -field exhibits odd symmetry and the $E_{z}\left(2\omega^{+}\right)$ -field shows even symmetry for the SRR-ITO coupled system. This even symmetric $E_{z}\left(2\omega^{+}\right)$ -field enables constructive polarization interference with the squared field $E_{z}^{2}\left(\omega^{+}\right)$ , leading to a larger integrand in Equation (1) and resulting in a 1218-fold enhancement in SHG compared to the nanorod-ITO coupled system.

**Figure 5:**
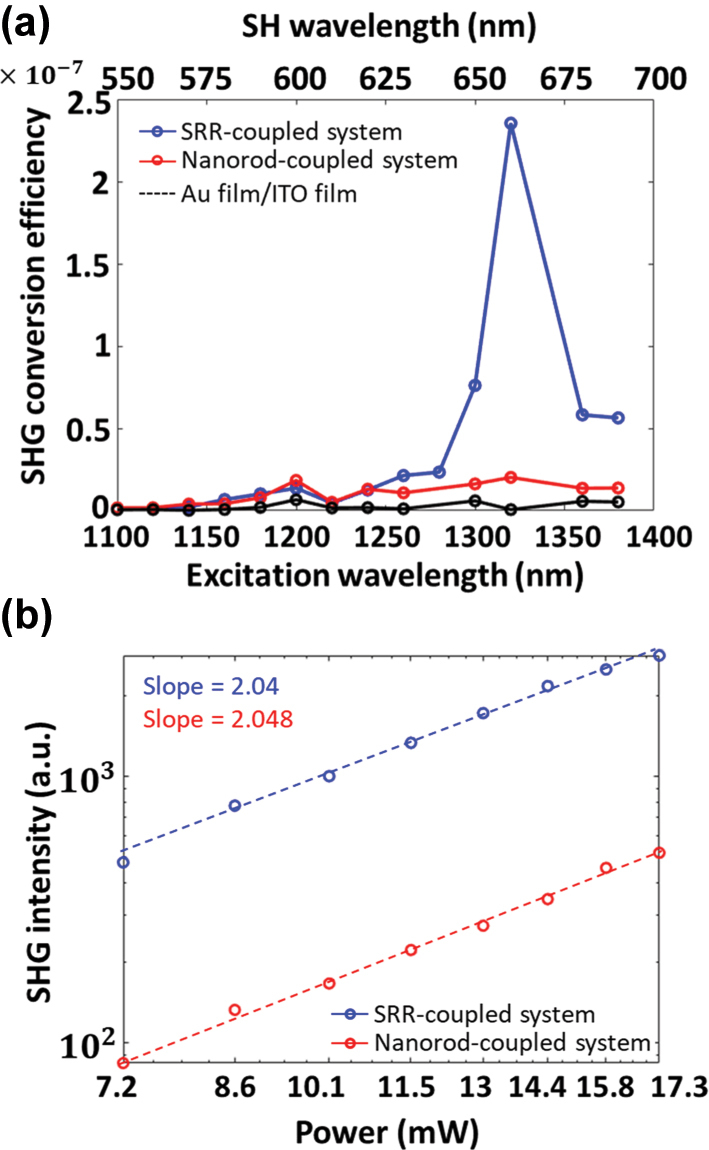
SHG performance of coupled systems. (a) Measured SHG conversion efficiency spectra for the SRR-ITO coupled system, the nanorod-ITO coupled system, and the Au /ITO film under an excitation wavelength range of 1,100–1,395 nm with a peak intensity of 4.6 GW/cm^2^. (b) Log–log plot of the measured SHG signals under varying excitation power. The dash lines indicate the fitting data with a second-order power dependence.

The SHG signals for the fabricated hybrid metasurfaces were then measured using in-house multiphoton microscopy with an excitation wavelength ranging from 1,100 to 1,395 nm (see details in section S3 of supplementary material). Figure 5a shows the measured SHG conversion efficiency, defined as $\eta_{SH}=\frac{P_{\text{SH}}}{P_{\text{in}}}$ , where *P*_in_ is input power and *P*_SH_ represents the collected SH power, for the SRR-ITO coupled system (blue dots), the nanorod-ITO coupled system (red dots), and the Au /ITO film (black dots). All measured data in the SHG conversion efficiency are obtained under normal incidence with an average power of 7.2 mW (measured after the objective lens, see details in Figure S1 of supplementary material), corresponding to a peak intensity of 4.6 GW/cm^2^ evaluated under a temporal pulse width of 200 fs, the repetition rate of 80 MHz, and the spot size of 3.5 μm for the fundamental beam. The maximal SHG conversion efficiency of 2.3 × 10^−7^ for the SRR-ITO coupled system is observed close to the excitation wavelength of hybridized ω^+^ resonance, corresponding to a one-order-of-magnitude enhancement compared to the nanorod-ITO coupled system and a two-order-of-magnitude enhancement compared to the Au/ITO film. The normalized conversion efficiency of the SRR-ITO coupled system, defined as $\zeta_{SH}=\frac{P_{\text{peak }-\text{ SH}}}{{P_{\text{peak }-\text{ in}}}^{2}}$ , where *P*_peak−SH_ is the peak SH power and *P*_peak−in_ is the peak pump power, reaches 2 × 10^−10^ *w* ^−1^. Finally, the SHG intensities for the coupled systems were measured at the excitation wavelength of 1,320 nm under varying excitation powers ranging from 7.2 to 17.3 mW (measured after the objective lens, see details in Figure S1 of supplementary material), as shown in Figure 5b. The SHG intensities follow a quadratic dependence on excitation power, with a slope of 2.04 and 2.05 for the SRR-ITO and the nanorod-ITO coupled systems, respectively, confirming that the observed nonlinear responses are indeed produced by SHG.

## 3 Summary and outlook

We investigate the coupling between a double-resonance SRR design and the ENZ mode on an ultrathin ITO film and their effects on SHG. To maximize the coupling efficiency, the AR of SRR structures in the uncoupled system is first optimized by employing the overlap integral, and we identified an AR of 0.3 achieves a double-resonance design that supports a MD resonance in the ITO’s ENZ region and a higher-order resonance at the SH frequency with a cross-polarization property, while the nanorod represents a single ED resonance within the ENZ region. The dominant *y*-polarized (*x*-polarized) SH response from the SRRs (nanorods) is consistent with the hydrodynamic model’s prediction of the SHG origin. Then, the large *χ* ^(2)^ ITO film is integrated to form the SRR-ITO and nanorod-ITO coupled systems. The strong coupling in both coupled systems result in two hybridized modes with a Rabi energy exceeding 200 meV and achieve a spectral overlap between the higher-order mode under y-polarized excitation and the SH frequency of *ω* ^+^ hybridized mode under x-polarized excitation. Near-field analysis of the SRR-ITO coupled system reveals that the even-symmetry of both the nonlinear polarization current $\chi^{\left(2\right)}E_{z}^{2}\left(\omega^{+}\right)$ and the linear *E*_*z*_(2ω^+^)-field leads to constructive polarization interference within the ITO layer. In contrast, the nanorod-ITO coupled system exhibits destructive interference due to a spatial symmetry mismatch between the $E_{z}^{2}\left(\omega^{+}\right)$ and the linear *E*_*z*_(2ω^+^)-field. As a result, the SRR-ITO coupled system achieves a three-order-magnitude enhancement in SHG efficiency compared to the nanorod-ITO coupled system, as predicted by the overlap integral analysis. The measured SHG of the SRR-ITO coupled system exhibits a peak SHG conversion efficiency of 2.3 × 10^−7^, which is one-order-magnitude (two-order-magnitude) enhancement compared to the nanorod-ITO coupled system (Au/ITO film). The high efficiency SH nonlinearity and the cross-polarization characteristics between the fundamental and SH signals for SRR-ITO coupled system open up new possibilities for developing advanced nonlinear photonic devices in integrated photonic circuits such as light sources and modulators.

## Supplementary Material

Comparison of state-of-the-art nonlinear metasurfaces for SHG; details of sample fabrication and linear and nonlinear optical characterization; linear optical properties of the uncoupled and coupled systems.

## Supplementary Material

Supplementary Material Details
