# Supplementary material for: Strong coupling of double resonance designs and epsilon-near-zero modes for mode-matching enhancement of second-harmonic generation
Source: Nanophotonics. 2025 Oct 23;14(25):4555–64. doi: 10.1515/nanoph-2025-0382 (PMC12714063; doi:10.1515/nanoph-2025-0382)
Supplement: Supplementary file 1 — Supplementary Material Details [file j_nanoph-2025-0382_suppl_001.docx]

Supplementary Material

**Strong Coupling of Double Resonance Designs and Epsilon-near-zero Modes for Mode-Matching Enhancement of Second-Harmonic Generation**

Ai-Yin Liu^1^, Chun-Hsiang Tseng^2^, Kuang-I Lin^3^, and Hui-Hsin Hsiao^1,2,*^

^1^Graduate Institute of Photonics and Optoelectronics, National Taiwan University, Taipei, 10617, Taiwan

^2^Department of Engineering Science and Ocean Engineering, National Taiwan University, Taipei 10617, Taiwan

^3^Core Facility Center, National Cheng Kung University, Tainan 70101, Taiwan

*E-mail: [hhhsiao@ntu.edu.tw](mailto:hhhsiao@ntu.edu.tw).

**Section S1:** **Comparison of state-of-the-art nonlinear metasurfaces for SHG**

Table 1 lists state-of-the-art nonlinear metasurfaces that employ either double-resonance designs or strong coupling mechanisms for the enhancement of SHG. While numerous double-resonance designs have been demonstrated for efficient SHG through the excited local field at both the fundamental and SH frequencies, only a few explicitly investigated the necessity of constructive interference between the nonlinear polarization currents and the linear electric-field at SH frequency for maximizing the SHG based on overlap integral. Noor *et. al.* investigated the constructive interference in a metal–insulator–metal sandwich structure, which requires oblique incidence to excite both the first- and second-order gap-plasmon modes. The SRR-ITO coupled system, on the other hand, achieves the constructive interference between the upper branch of hybridized mode ($\omega^{+}$) and the higher-order mode under normal incidence.

In addition, strong coupling between plasmonic or dielectric nanostructures and the ENZ mode of ultrathin ITO films has been extensively studied for various nonlinear processes, including Kerr effects and nonlinear signal up- or down-conversion. Most prior works, however, focus on overlapping a single resonant mode with the ENZ wavelength of ITO films. Only a few research works have explored the coupling of double-resonance designs with other mechanisms such as excitons in dye molecules (Ref. 1 in Table 1) or the ENZ mode of ITO films (our work).

**Table 1**

**
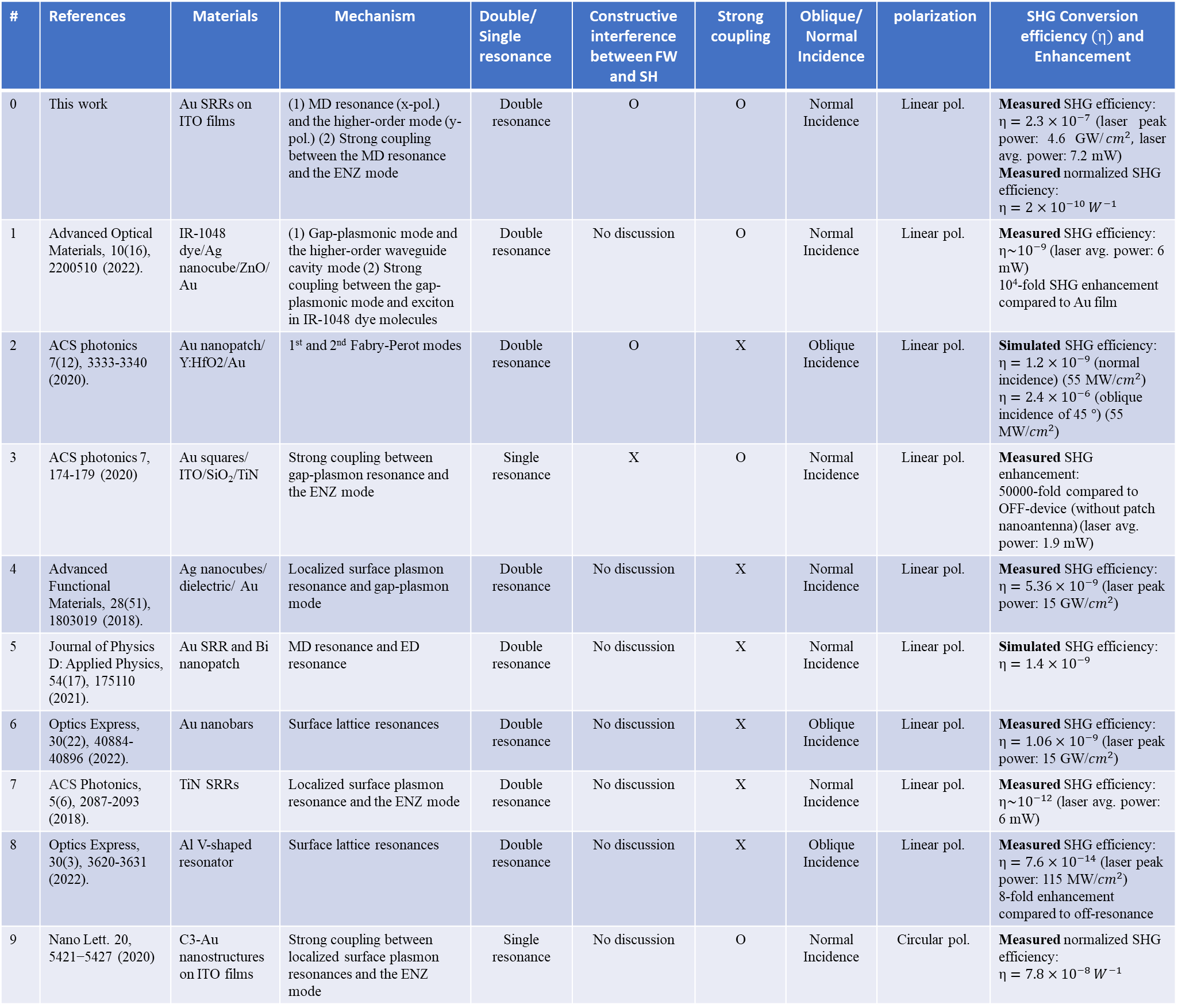
**

MD: magnetic dipole; ED: electric dipole; ENZ: epsilon-near-zero

**Section S2: Fabrication**

To fabricate the hybrid metasuface, the electron beam resist ZEP520A and a conductive Espacer layer were first spin-coated on the ITO substrate. Electron beam lithography was then performed using an ELS-7000 system, operating at an acceleration voltage of 100 kV and a beam current of 30 pA, to define the nanostructures within a 150×150 μm^2^ area. After exposure, the Espacer layer was removed by rinsing with deionized water, followed by development in a ZED-N50 solution. A 5 nm-thick chromium adhesion layer was then deposited, followed by a 50 nm-thick gold layer using electron beam evaporation. Finally, the lift-off process was carried out using N,N-dimethylacetamide (ZDMAC), completing the sample fabrication.

**Section S3: Optical characterization**

*Linear optical measurements*

The transmission spectra of the fabricated samples were measured using an in-housed built microscope (10× Infinity Corrected objective lens, numerical aperture (NA) = 0.25) equipped with a near-infrared spectrometer (Ocean optics, InGaAs detector). A halogen lamp was used as the illumination source, generating a focal spot of approximately 100 μm on the sample surface. A CCD camera was integrated into the system to accurately identify the fabricated structures.

*Nonlinear optical measurements*

The SHG spectra were conducted by custom-built multiphoton excitation microscope as illustrated in Figure S1. The excitation light was produced by an optical parametric oscillator (OPO) pumped by a mode-locked Ti:sapphire laser (Coherent Chameleon Vision II), offering a tunable wavelength range between 1100 nm and 1395 nm. The laser operated at an 80 MHz repetition rate, delivering ultrafast pulses with a duration of 200 fs. The beam was tightly focused onto the sample through a 10× objective with a NA of 0.45, achieving a focal spot of approximately 3 μm. The backscattered SH signal was detected by four photomultiplier tubes, each fitted with band-pass filters to selectively capture the target harmonic signal while suppressing unwanted spectral contributions [1].


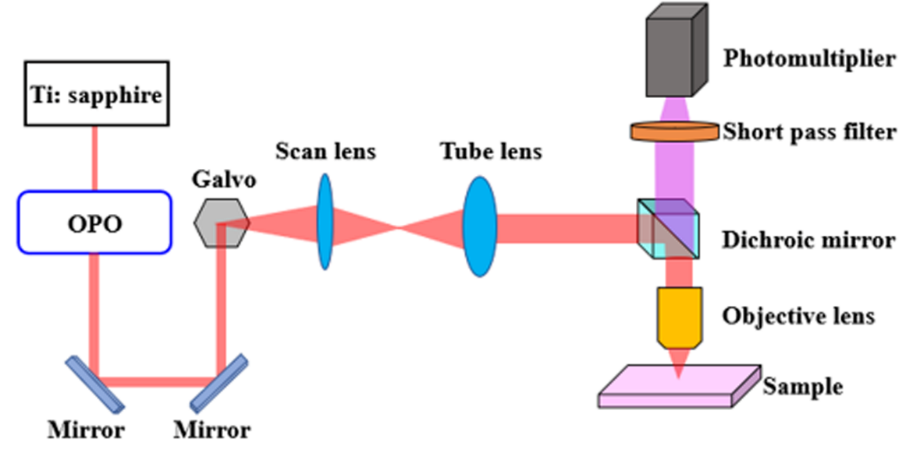


**Figure S1:** The scheme of the optical setup for THG measurement [1].


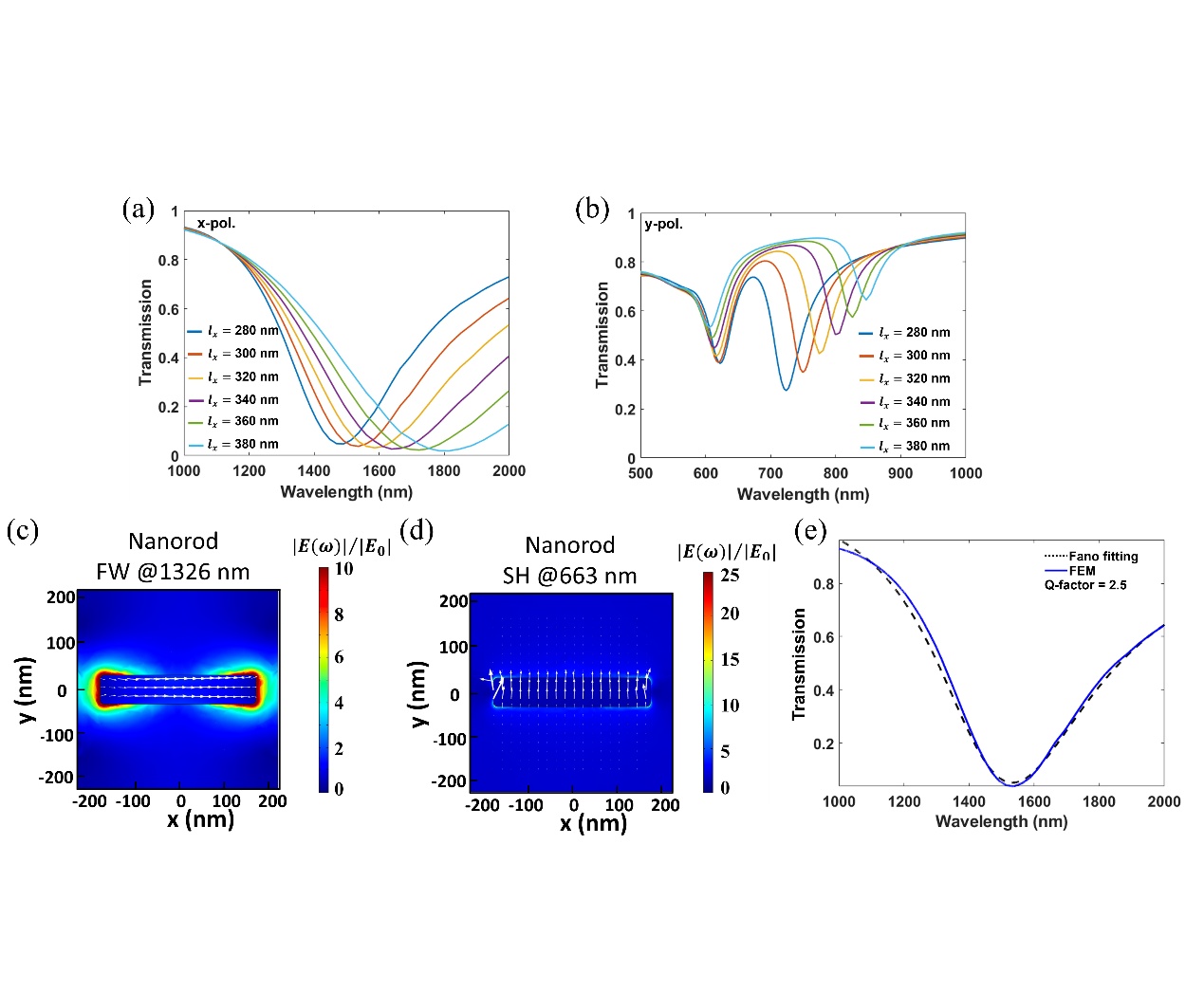
**Section S4:** **Linear optical properties of the uncoupled system**

**Figure S2**. Simulated transmission spectra of SRRs with varying aspect ratios under (a) *x*-polarized and (b) *y*-polarized light illumination. Electric field distributions of the nanorod (c) at the fundamental wavelength under *x*-polarized excitation and (d) at the SH wavelength under *y*-polarized excitation. White arrows indicate the surface current distributions. (e) Simulated spectrum of the optimized SRR (blue curve) and the Fano line-shape fitting curve (dashed curve).

The calculated transmission spectra are fitted using the Fano line-shape function, as described by equation (6) in the main text. Figure S2e presents the fitting results for the SRR arrays (without the ITO film) with $l_{x}$ = 300 nm (aspect ratio = 0.3). The fitting spectrum (black dashed curve) exhibits excellent agreement with the simulated result (blue curve). The corresponding Q-factor is evaluated $Q_{tot}=\frac{\omega_{0}}{2\gamma_{tot}}$, yielding a value of 2.5.

**Section S5:** **Optical properties of the coupled system**


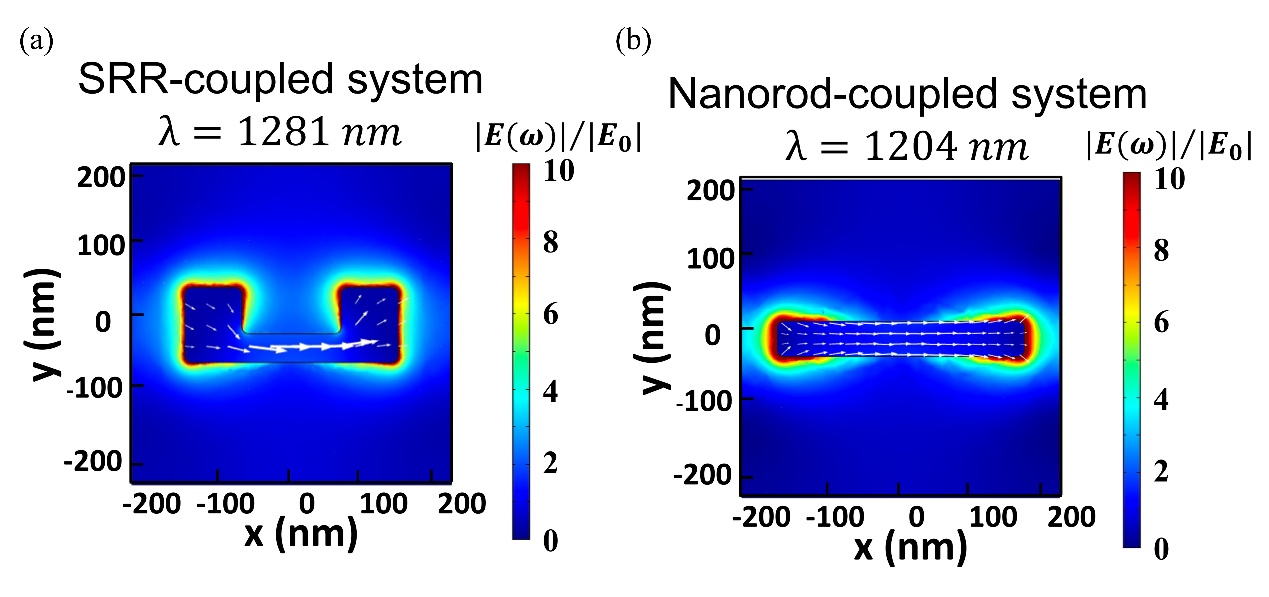


**Figure S3**. Electric field distributions at the respective $\omega^{+}$ resonance for (a) the SRR-ITO and (b) the nanorod-ITO coupled systems, respectively.

**References**

1. A.-Y. Liu, J.-C. Hsieh, K.-I. Lin, S. H. Tseng, and H.-H. Hsiao, "Third Harmonic Generation Enhanced by Generalized Kerker Condition in All-Dielectric Metasurfaces," Advanced Optical Materials **11**, 2300526 (2023).
